# Risks to Health and Well-Being From Radio-Frequency Radiation Emitted by Cell Phones and Other Wireless Devices

**DOI:** 10.3389/fpubh.2019.00223

**Published:** 2019-08-13

**Authors:** Anthony B. Miller, Margaret E. Sears, L. Lloyd Morgan, Devra L. Davis, Lennart Hardell, Mark Oremus, Colin L. Soskolne

**Affiliations:** ^1^Dalla Lana School of Public Health, University of Toronto, Toronto, ON, Canada; ^2^Ottawa Hospital Research Institute, Prevent Cancer Now, Ottawa, ON, Canada; ^3^Environmental Health Trust, Teton Village, WY, United States; ^4^The Environment and Cancer Research Foundation, Örebro, Sweden; ^5^School of Public Health and Health Systems, University of Waterloo, Waterloo, ON, Canada; ^6^School of Public Health, University of Alberta, Edmonton, AB, Canada; ^7^Health Research Institute, University of Canberra, Canberra, ACT, Australia

**Keywords:** brain cancer, electromagnetic hypersensitivity, glioma, non-cancer outcomes, policy recommendations, radiofrequency fields, child development, acoustic neuroma

## Abstract

Radiation exposure has long been a concern for the public, policy makers, and health researchers. Beginning with radar during World War II, human exposure to radio-frequency radiation^1^ (RFR) technologies has grown substantially over time. In 2011, the *International Agency for Research on Cancer* (IARC) reviewed the published literature and categorized RFR as a “possible” (Group 2B) human carcinogen. A broad range of adverse human health effects associated with RFR have been reported since the IARC review. In addition, three large-scale carcinogenicity studies in rodents exposed to levels of RFR that mimic lifetime human exposures have shown significantly increased rates of Schwannomas and malignant gliomas, as well as chromosomal DNA damage. Of particular concern are the effects of RFR exposure on the developing brain in children. Compared with an adult male, a cell phone held against the head of a child exposes deeper brain structures to greater radiation doses per unit volume, and the young, thin skull's bone marrow absorbs a roughly 10-fold higher local dose. Experimental and observational studies also suggest that men who keep cell phones in their trouser pockets have significantly lower sperm counts and significantly impaired sperm motility and morphology, including mitochondrial DNA damage. Based on the accumulated evidence, we recommend that IARC re-evaluate its 2011 classification of the human carcinogenicity of RFR, and that WHO complete a systematic review of multiple other health effects such as sperm damage. In the interim, current knowledge provides justification for governments, public health authorities, and physicians/allied health professionals to warn the population that having a cell phone next to the body is harmful, and to support measures to reduce all exposures to RFR.

## Introduction

We live in a generation that relies heavily on technology. Whether for personal use or work, wireless devices, such as cell phones, are commonly used around the world, and exposure to radio-frequency radiation (RFR) is widespread, including in public spaces (1, 2).

In this review, we address the current scientific evidence on health risks from exposure to RFR, which is in the non-ionizing frequency range. We focus here on human health effects, but also note evidence that RFR can cause physiological and/or morphological effects on bees, plants and trees (3–5).

We recognize a diversity of opinions on the potential adverse effects of RFR exposure from cell or mobile phones and other wireless transmitting devices (WTDs) including cordless phones and Wi-Fi. The paradigmatic approach in cancer epidemiology, which considers the body of epidemiological, toxicological, and mechanistic/cellular evidence when assessing causality, is applied.

## Carcinogenicity

Since 1998, the *International Commission on Non-Ionizing Radiation Protection* (ICNIRP) has maintained that no evidence of adverse biological effects of RFR exist, other than tissue heating at exposures above prescribed thresholds (6).

In contrast, in 2011, an expert working group of the *International Agency for Research on Cancer* (IARC) categorized RFR emitted by cell phones and other WTDs as a Group 2B (“possible”) human carcinogen (7).

Since the IARC categorization, analyses of the large international Interphone study, a series of studies by the Hardell group in Sweden, and the French CERENAT case-control studies, signal increased risks of brain tumors, particularly with ipsilateral use (8). The largest case-control studies on cell phone exposure and glioma and acoustic neuroma demonstrated significantly elevated risks that tended to increase with increasing latency, increasing cumulative duration of use, ipsilateral phone use, and earlier age at first exposure (8).

Pooled analyses by the Hardell group that examined risk of glioma and acoustic neuroma stratified by age at first exposure to cell phones found the highest odds ratios among those first exposed before age 20 years (9–11). For glioma, first use of cell phones before age 20 years resulted in an odds ratio (OR) of 1.8 (95% confidence interval [CI] 1.2–2.8). For ipsilateral use, the OR was 2.3 (CI 1.3-4.2); contralateral use was 1.9 (CI 0.9-3.7). Use of cordless phone before age 20 yielded OR 2.3 (CI 1.4–3.9), ipsilateral OR 3.1 (CI 1.6–6.3) and contralateral use OR 1.5 (CI 0.6–3.8) (9).

Although Karipidis et al. (12) and Nilsson et al. (13) found no evidence of an increased incidence of gliomas in recent years in Australia and Sweden, respectively, Karipidis et al. (12) only reported on brain tumor data for ages 20–59 and Nilsson et al. (13) failed to include data for high grade glioma. In contrast, others have reported evidence that increases in specific types of brain tumors seen in laboratory studies are occurring in Britain and the US:

- The incidence of neuro-epithelial brain cancers has significantly increased in all children, adolescent, and young adult age groupings from birth to 24 years in the United States (14, 15).
- A sustained and statistically significant rise in glioblastoma multiforme across all ages has been described in the UK (16).

The incidence of several brain tumors are increasing at statistically significant rates, according to the 2010–2017 *Central Brain Tumor Registry of the U.S*. (CBTRUS) dataset (17).

- There was a significant increase in incidence of radiographically diagnosed tumors of the pituitary from 2006 to 2012 (APC = 7.3% [95% CI: 4.1%, 10.5%]), with no significant change in incidence from 2012 to 2015 (18).
- Meningioma rates have increased in all age groups from 15 through 85+ years.
- Nerve sheath tumor (Schwannoma) rates have increased in all age groups from age 20 through 84 years.
- Vestibular Schwannoma rates, as a percentage of nerve sheath tumors, have also increased from 58% in 2004 to 95% in 2010-2014.

Epidemiological evidence was subsequently reviewed and incorporated in a meta-analysis by Röösli et al. (19). They concluded that overall, epidemiological evidence does not suggest increased brain or salivary gland tumor risk with mobile phone (MP) use, although the authors admitted that some uncertainty remains regarding long latency periods (>15 years), rare brain tumor subtypes, and MP usage during childhood. Of concern is that these analyses included cohort studies with poor exposure classification (20).

In epidemiological studies, recall bias can play a substantial role in the attenuation of odds ratios toward the null hypothesis. An analysis of data from one large multicenter case-control study of RFR exposure, did not find that recall bias was an issue (21). In another multi-country study it was found that young people can recall phone use moderately well, with recall depending on the amount of phone use and participants' characteristics (22). With less rigorous querying of exposure, prospective cohort studies are unfortunately vulnerable to exposure misclassification and imprecision in identifying risk from rare events, to the point that negative results from such studies are misleading (8, 23).

Another example of disparate results from studies of different design focuses on prognosis for patients with gliomas, depending upon cell phone use. A Swedish study on glioma found lower survival in patients with glioblastoma associated with long term use of wireless phones (24). Ollson et al. (25), however, reported no indication of reduced survival among glioblastoma patients in Denmark, Finland and Sweden with a history of mobile phone use (ever regular use, time since start of regular use, cumulative call time overall or in the last 12 months) relative to no or non-regular use. Notably, Olsson et al. (25) differed from Carlberg and Hardell (24) in that the study did not include use of cordless phones, used shorter latency time and excluded patients older than 69 years. Furthermore, a major shortcoming was that patients with the worst prognosis were excluded, as in Finland inoperable cases were excluded, all of which would bias the risk estimate toward unity.

In the interim, three large-scale toxicological (animal carcinogenicity) studies support the human evidence, as do modeling, cellular and DNA studies identifying vulnerable sub-groups of the population.

The *U.S. National Toxicology Program* (NTP) (National Toxicology Program (26, 27) has reported significantly increased incidence of glioma and malignant Schwannoma (mostly on the nerves on the heart, but also additional organs) in large animal carcinogenicity studies with exposure to levels of RFR that did not significantly heat tissue. Multiple organs (e.g., brain, heart) also had evidence of DNA damage. Although these findings have been dismissed by the ICNIRP (28), one of the key originators of the NTP study has refuted the criticisms (29).

A study by Italy's Ramazzini Institute has evaluated lifespan environmental exposure of rodents to RFR, as generated by 1.8 GHz GSM antennae of cell phone radio base stations. Although the exposures were 60 to 6,000 times lower than those in the NTP study, statistically significant increases in Schwannomas of the heart in male rodents exposed to the highest dose, and Schwann-cell hyperplasia in the heart in male and female rodents were observed (30). A non-statistically significant increase in malignant glial tumors in female rodents also was detected. These findings with far field exposure to RFR are consistent with and reinforce the results of the NTP study on near field exposure. Both reported an increase in the incidence of tumors of the brain and heart in RFR-exposed Sprague-Dawley rats, which are tumors of the same histological type as those observed in some epidemiological studies on cell phone users.

Further, in a 2015 animal carcinogenicity study, tumor promotion by exposure of mice to RFR at levels below exposure limits for humans was demonstrated (31). Co-carcinogenicity of RFR was also demonstrated by Soffritti and Giuliani (32) who examined both power-line frequency magnetic fields as well as 1.8 GHz modulated RFR. They found that exposure to Sinusoidal-50 Hz Magnetic Field (S-50 Hz MF) combined with acute exposure to gamma radiation or to chronic administration of formaldehyde in drinking water induced a significantly increased incidence of malignant tumors in male and female Sprague Dawley rats. In the same report, preliminary results indicate higher incidence of malignant Schwannoma of the heart after exposure to RFR in male rats. Given the ubiquity of many of these co-carcinogens, this provides further evidence to support the recommendation to reduce the public's exposure to RFR to as low as is reasonably achievable.

Finally, a case series highlights potential cancer risk from cell phones carried close to the body. West et al. (33) reported four “extraordinary” multifocal breast cancers that arose directly under the antennae of the cell phones habitually carried within the bra, on the sternal side of the breast (the opposite of the norm). We note that case reports can point to major unrecognized hazards and avenues for further investigation, although they do not usually provide direct causal evidence.

In a study of four groups of men, of which one group did not use mobile phones, it was found that DNA damage indicators in hair follicle cells in the ear canal were higher in the RFR exposure groups than in the control subjects. In addition, DNA damage increased with the daily duration of exposure (34).

Many profess that RFR cannot be carcinogenic as it has insufficient energy to cause direct DNA damage. In a review, Vijayalaxmi and Prihoda (35) found some studies suggested significantly increased damage in cells exposed to RF energy compared to unexposed and/or sham-exposed control cells, others did not. Unfortunately, however, in grading the evidence, these authors failed to consider baseline DNA status or the fact that genotoxicity has been poorly predicted using tissue culture studies (36). As well funding, a strong source of bias in this field of enquiry, was not considered (37).

## Children and Reproduction

As a result of rapid growth rates and the greater vulnerability of developing nervous systems, the long-term risks to children from RFR exposure from cell phones and other WTDs are expected to be greater than those to adults (38). By analogy with other carcinogens, longer opportunities for exposure due to earlier use of cell phones and other WTDs could be associated with greater cancer risks in later life.

Modeling of energy absorption can be an indicator of potential exposure to RFR. A study modeling the exposure of children 3–14 years of age to RFR has indicated that a cell phone held against the head of a child exposes deeper brain structures to roughly double the radiation doses (including fluctuating electrical and magnetic fields) per unit volume than in adults, and also that the marrow in the young, thin skull absorbs a roughly 10-fold higher local dose than in the skull of an adult male (39). Thus, pediatric populations are among the most vulnerable to RFR exposure.

The increasing use of cell phones in children, which can be regarded as a form of addictive behavior (40), has been shown to be associated with emotional and behavioral disorders. Divan et al. (41) studied 13,000 mothers and children and found that prenatal exposure to cell phones was associated with behavioral problems and hyperactivity in children. A subsequent Danish study of 24,499 children found a 23% increased odds of emotional and behavioral difficulties at age 11 years among children whose mothers reported any cell phone use at age 7 years, compared to children whose mothers reported no use at age 7 years (42). A cross-sectional study of 4,524 US children aged 8–11 years from 20 study sites indicated that shorter screen time and longer sleep periods independently improved child cognition, with maximum benefits achieved with low screen time and age-appropriate sleep times (43). Similarly, a cohort study of Swiss adolescents suggested a potential adverse effect of RFR on cognitive functions that involve brain regions mostly exposed during mobile phone use (44). Sage and Burgio et al. (45) posit that epigenetic drivers and DNA damage underlie adverse effects of wireless devices on childhood development.

RFR exposure occurs in the context of other exposures, both beneficial (e.g., nutrition) and adverse (e.g., toxicants or stress). Two studies identified that RFR potentiated adverse effects of lead on neurodevelopment, with higher maternal use of mobile phones during pregnancy [1,198 mother-child pairs, (46)] and Attention Deficit Hyper-activity Disorder (ADHD) with higher cell phone use and higher blood lead levels, in 2,422 elementary school children (47).

A study of Mobile Phone Base Station Tower settings adjacent to school buildings has found that high exposure of male students to RFR from these towers was associated with delayed fine and gross motor skills, spatial working memory, and attention in adolescent students, compared with students who were exposed to low RFR (48). A recent prospective cohort study showed a potential adverse effect of RFR brain dose on adolescents' cognitive functions including spatial memory that involve brain regions exposed during cell phone use (44).

In a review, Pall (49) concluded that various non-thermal microwave EMF exposures produce diverse neuropsychiatric effects. Both animal research (50–52) and human studies of brain imaging research (53–56) indicate potential roles of RFR in these outcomes.

Male fertility has been addressed in cross-sectional studies in men. Associations between keeping cell phones in trouser pockets and lower sperm quantity and quality have been reported (57). Both *in vivo* and *in vitro* studies with human sperm confirm adverse effects of RFR on the testicular proteome and other indicators of male reproductive health (57, 58), including infertility (59). Rago et al. (60) found significantly altered sperm DNA fragmentation in subjects who use mobile phones for more than 4 h/day and in particular those who place the device in the trousers pocket. In a cohort study, Zhang et al. (61) found that cell phone use may negatively affect sperm quality in men by decreasing the semen volume, sperm concentration, or sperm count, thus impairing male fertility. Gautam et al. (62) studied the effect of 3G (1.8–2.5 GHz) mobile phone radiation on the reproductive system of male Wistar rats. They found that exposure to mobile phone radiation induces oxidative stress in the rats which may lead to alteration in sperm parameters affecting their fertility.

## Related Observations, Implications and Strengths of Current Evidence

An extensive review of numerous published studies confirms non-thermally induced biological effects or damage (e.g., oxidative stress, damaged DNA, gene and protein expression, breakdown of the blood-brain barrier) from exposure to RFR (63), as well as adverse (chronic) health effects from long-term exposure (64). Biological effects of typical population exposures to RFR are largely attributed to fluctuating electrical and magnetic fields (65–67).

Indeed, an increasing number of people have developed constellations of symptoms attributed to exposure to RFR (e.g., headaches, fatigue, appetite loss, insomnia), a syndrome termed *Microwave Sickness* or *Electro-Hyper-Sensitivity* (EHS) (68–70).

Causal inference is supported by consistency between epidemiological studies of the effects of RFR on induction of human cancer, especially glioma and vestibular Schwannomas, and evidence from animal studies (8). The combined weight of the evidence linking RFR to public health risks includes a broad array of findings: experimental biological evidence of non-thermal effects of RFR; concordance of evidence regarding carcinogenicity of RFR; human evidence of male reproductive damage; human and animal evidence of developmental harms; and limited human and animal evidence of potentiation of effects from chemical toxicants. Thus, diverse, independent evidence of a potentially troubling and escalating problem warrants policy intervention.

## Challenges to Research, From Rapid Technological Advances

Advances in RFR-related technologies have been and continue to be rapid. Changes in carrier frequencies and the growing complexity of modulation technologies can quickly render “yesterdays” technologies obsolete. This rapid obsolescence restricts the amount of data on human RFR exposure to particular frequencies, modulations and related health outcomes that can be collected during the lifespan of the technology in question.

Epidemiological studies with adequate statistical power must be based upon large numbers of participants with sufficient latency and intensity of exposure to specific technologies. Therefore, a lack of epidemiological evidence does not necessarily indicate an absence of effect, but rather an inability to study an exposure for the length of time necessary, with an adequate sample size and unexposed comparators, to draw clear conclusions. For example, no case-control study has been published on fourth generation (4G; 2–8 GHz) Long-term Evolution (LTE) modulation, even though the modulation was introduced in 2010 and achieved a 39% market share worldwide by 2018 (71).

With this absence of human evidence, governments must require large-scale animal studies (or other appropriate studies of indicators of carcinogenicity and other adverse health effects) to determine whether the newest modulation technologies incur risks, prior to release into the marketplace. Governments should also investigate short-term impacts such as insomnia, memory, reaction time, hearing and vision, especially those that can occur in children and adolescents, whose use of wireless devices has grown exponentially within the past few years.

The Telecom industry's fifth generation (5G) wireless service will require the placement of many times more small antennae/cell towers close to all recipients of the service, because solid structures, rain and foliage block the associated millimeter wave RFR (72). Frequency bands for 5G are separated into two different frequency ranges. Frequency Range 1 (FR1) includes sub-6 GHz frequency bands, some of which are bands traditionally used by previous standards, but has been extended to cover potential new spectrum offerings from 410 to 7,125 MHz. Frequency Range 2 (FR2) includes higher frequency bands from 24.25 to 52.6 GHz. Bands in FR2 are largely of millimeter wave length, these have a shorter range but a higher available bandwidth than bands in the FR1. 5G technology is being developed as it is also being deployed, with large arrays of directional, steerable, beam-forming antennae, operating at higher power than previous technologies. 5G is not stand-alone—it will operate and interface with other (including 3G and 4G) frequencies and modulations to enable diverse devices under continual development for the “internet of things,” driverless vehicles and more (72).

Novel 5G technology is being rolled out in several densely populated cities, although potential chronic health or environmental impacts have not been evaluated and are not being followed. Higher frequency (shorter wavelength) radiation associated with 5G does not penetrate the body as deeply as frequencies from older technologies although its effects may be systemic (73, 74). The range and magnitude of potential impacts of 5G technologies are under-researched, although important biological outcomes have been reported with millimeter wavelength exposure. These include oxidative stress and altered gene expression, effects on skin and systemic effects such as on immune function (74). *In vivo* studies reporting resonance with human sweat ducts (73), acceleration of bacterial and viral replication, and other endpoints indicate the potential for novel as well as more commonly recognized biological impacts from this range of frequencies, and highlight the need for research before population-wide continuous exposures.

## Gaps in Applying Current Evidence

Current exposure limits are based on an assumption that the only adverse health effect from RFR is heating from short-term (acute), time-averaged exposures (75). Unfortunately, in some countries, notably the US, scientific evidence of the potential hazards of RFR has been largely dismissed (76). Findings of carcinogenicity, infertility and cell damage occurring at daily exposure levels—within current limits—indicate that existing exposure standards are not sufficiently protective of public health. Evidence of carcinogenicity alone, such as that from the NTP study, should be sufficient to recognize that current exposure limits are inadequate.

Public health authorities in many jurisdictions have not yet incorporated the latest science from the U.S. NTP or other groups. Many cite 28-year old guidelines by the *Institute of Electrical and Electronic Engineers* which claimed that “Research on the effects of chronic exposure and speculations on the biological significance of non-thermal interactions have not yet resulted in any meaningful basis for alteration of the standard” (77)^2^.

Conversely, some authorities have taken specific actions to reduce exposure to their citizens (78), including testing and recalling phones that exceed current exposure limits.

While we do not know how risks to individuals from using cell phones may be offset by the benefits to public health of being able to summon timely health, fire and police emergency services, the findings reported above underscore the importance of evaluating potential adverse health effects from RFR exposure, and taking pragmatic, practical actions to minimize exposure.

We propose the following considerations to address gaps in the current body of evidence:

- As many claim that we should by now be seeing an increase in the incidence of brain tumors if RFR causes them, ignoring the increases in brain tumors summarized above, a detailed evaluation of age-specific, location-specific trends in the incidence of gliomas in many countries is warranted.
- Studies should be designed to yield the strongest evidence, most efficiently:
  ➢ Population-based case-control designs can be more statistically powerful to determine relationships with rare outcomes such as glioma, than cohort studies. Such studies should explore the relationship between energy absorption (SAR^3^), duration of exposure, and adverse outcomes, especially brain cancer, cardiomyopathies and abnormal cardiac rythms, hematologic malignancies, thyroid cancer.
  ➢ Cohort studies are inefficient in the study of rare outcomes with long latencies, such as glioma, because of cost-considerations relating to the follow-up required of very large cohorts needed for the study of rare outcomes. In addition, without continual resource-consuming follow-up at frequent intervals, it is not possible to ascertain ongoing information about changing technologies, uses (e.g., phoning vs. texting or accessing the Internet) and/or exposures.
  ➢ Cross-sectional studies comparing high-, medium-, and low-exposure persons may yield hypothesis-generating information about a range of outcomes relating to memory, vision, hearing, reaction-time, pain, fertility, and sleep patterns.
- Exposure assessment is poor in this field, with very little fine-grained detail as to frequencies and modulations, doses and dose rates, and peak exposures, particularly over the long-term. Solutions such as wearable meters and phone apps have not yet been incorporated in large-scale research.
- Systematic reviews on the topic could use existing databases of research reports, such as the one created by *Oceania Radiofrequency Science Advisory Association* (79) or EMF Portal (80), to facilitate literature searches.
- Studies should be conducted to determine appropriate locations for installation of antennae and other broadcasting systems; these studies should include examination of biomarkers of inflammation, genotoxicity, and other health indicators in persons who live at different radiuses around these installations. This is difficult to study in the general population because many people's greatest exposure arises from their personal devices.
- Further work should be undertaken to determine the distance that wireless technology antennae should be kept away from humans to ensure acceptable levels of safety, distinguishing among a broad range of sources (e.g., from commercial transmitters to Bluetooth devices), recognizing that exposures fall with the inverse of the square of the distance (The inverse-square law specifies that intensity is inversely proportional to the square of the distance from the source of radiation). The effective radiated power from cell towers needs to be regularly measured and monitored.

## Policy Recommendations Based on the Evidence to Date

At the time of writing, a total of 32 countries or governmental bodies within these countries^4^ have issued policies and health recommendations concerning exposure to RFR (78). Three U.S. states have issued advisories to limit exposure to RFR (81–83) and the *Worcester Massachusetts Public Schools* (84) voted to post precautionary guidelines on Wi-Fi radiation on its website. In France, Wi-Fi has been removed from pre-schools and ordered to be shut off in elementary schools when not in use, and children aged 16 years or under are banned from bringing cell phones to school (85). Because the national test agency found 9 out of 10 phones exceeded permissible radiation limits, France is also recalling several million phones.

We therefore recommend the following:

1. Governmental and institutional support of data collection and analysis to monitor potential links between RFR associated with wireless technology and cancers, sperm, the heart, the nervous system, sleep, vision and hearing, and effects on children.
2. Further dissemination of information regarding potential health risk information that is in wireless devices and manuals is necessary to respect users' *Right To Know*. Cautionary statements and protective measures should be posted on packaging and at points of sale. Governments should follow the practice of France, Israel and Belgium and mandate labeling, as for tobacco and alcohol.
3. Regulations should require that any WTD that could be used or carried directly against the skin (e.g., a cell phone) or in close proximity (e.g., a device being used on the lap of a small child) be tested appropriately as used, and that this information be prominently displayed at point of sale, on packaging, and both on the exterior and within the device.
4. IARC should convene a new working group to update the categorization of RFR, including current scientific findings that highlight, in particular, risks to youngsters of subsequent cancers. We note that an IARC Advisory Group has recently recommended that RFR should be re-evaluated by the IARC Monographs program with high priority.
5. The World Health Organization (WHO) should complete its long-standing RFR systematic review project, using strong modern scientific methods. National and regional public health authorities similarly need to update their understanding and to provide adequate precautionary guidance for the public to minimize potential health risks.
6. Emerging human evidence is confirming animal evidence of developmental problems with RFR exposure during pregnancy. RFR sources should be avoided and distanced from expectant mothers, as recommended by physicians and scientists (babysafeproject.org).
7. Other countries should follow France, limiting RFR exposure in children under 16 years of age.
8. Cell towers should be distanced from homes, daycare centers, schools, and places frequented by pregnant women, men who wish to father healthy children, and the young.

Specific examples of how the health policy recommendations above, invoking the Precautionary Principle, might be practically applied to protect public health, are provided in the Annex.

## Author Contributions

All authors listed have made a substantial, direct and intellectual contribution to the work, and approved it for publication.

### Conflict of Interest Statement

The authors declare that this manuscript was drafted in the absence of any commercial or financial relationships that could be construed as a potential conflict of interest, although subsequent to its preparation, DD became a consultant to legal counsel representing persons with glioma attributed to radiation from cell phones.

## Figures and Tables

**Table:** 

ALARA	As Low a level As Reasonably Achievable
CBTRUS	Central Brain Tumor Registry of the United States
CI	Confidence Interval
EMR	Electro Magnetic Radiation
IARC	International Agency for Research on Cancer
ICNIRP	International Commission on Non-Ionizing Radiation Protection
INEP	International Network for Epidemiology in Policy
LTE	Long-Term Evolution modulation
NTP	U.S. National Toxicology Program
OR	Odds Ratio
RFR	Radio-Frequency Radiation
SAR	Specific Absorption Rate
WTD	Wireless Transmitting Device

## Footnotes

^2^The FCC adopted the IEEE C95.1 1991 standard in 1996.

^3^When necessary, SAR values should be adjusted for age of child in W/kg.

^4^Argentina, Australia, Austria, Belgium, Canada, Chile, Cyprus, Denmark, European Environmental Agency, European Parliament, Finland, France, French Polynesia, Germany, Greece, Italy, India, Ireland, Israel, Namibia, New Zealand, Poland, Romania, Russia, Singapore, Spain, Switzerland, Taiwan, Tanzania, Turkey, United Kingdom, United States.

## Annex: Examples of Actions for Reducing RFR Exposure

1. Focus actions for reducing exposure to RFR on pregnant women, infants, children and adolescents, as well as males who might wish to become fathers.
2. Reduce, as much as possible, the extent to which infants and young children are exposed to RFR from Wi-Fi-enabled devices such as baby monitors, wearable devices, cell phones, tablets, etc.
3. Avoid placing cell towers and small cell antennae close to schools and homes pending further research and revision of the existing exposure limits. In schools, homes and the workplace, cable or optical fiber connections to the Internet are preferred. Wi-Fi routers in schools and daycares/kindergartens should be strongly discouraged and programs instituted to provide Internet access via cable or fiber.
4. Ensure that WTDs minimize radiation by transmitting only when necessary, and as infrequently as is feasible. Examples include transmitting only in response to a signal (e.g., accessing a router or querying a device, a cordless phone handset being turned on, or voice or motion activation). Prominent, visible power switches are needed to ensure that WTDs can be easily turned on only when needed, and off when not required (e.g., Wi-Fi when sleeping).
5. Lower permitted power densities in close proximity to fixed-site antennae, from “occupational” limits to exposure limits for the general public.
6. Update current exposure limits to be protective against the non-thermal effects of RFR. Such action should be taken by all heath ministries and public health agencies, as well as industry regulatory bodies. Exposure limits should be based on measurements of RFR levels related to biological effects (2).
7. Ensure that advisories relating to cell phone use are placed in such a way that purchasers can find them easily, similar to the Berkeley Cell Phone “Right to Know” Ordinance (86).
8. Advise the public that texting and speaker mode are preferable to holding cell phones to the ear. Alternatively, use hands-free accessories for cell phones, including air tube headsets that interrupt the transmission of RFR.
9. When possible, keep cell phones away from the body (e.g., on a nearby desk, in a purse or bag, or on a mounted hands-free accessory in motor vehicles).
10. Delay the widespread implementation of 5G (and any other new technology) until studies can be conducted to assess safety. This includes a wide range of household and community-wide infrastructure WTDs and self-driving vehicles, as well as the building of 5G minicells.
11. Fiber-optic connections for the Internet should be made available to every home, office, school, warehouse and factory, when and where possible.
